# Stigmasterol Attenuates Triple-negative Breast Cancer Stem Cell Properties by Inhibiting JAK3

**DOI:** 10.7150/jca.94822

**Published:** 2025-02-03

**Authors:** Ruijuan Zhou, Yuzhu Zhang, Leqin Xu, Yang Sun

**Affiliations:** 1 Department of Chest and Breast Surgery, Xiamen Hospital of Traditional Chinese Medicine, Fujian University of Traditional Chinese Medicine, Xiamen, China.; 2 Breast Department, Guangdong Provincial Hospital of Chinese Medicine, The Second Affiliated Hospital of Guangzhou University of Chinese Medicine, Guangzhou, China.

**Keywords:** Stigmasterol, Breast cancer, CSCs, JAK3

## Abstract

**Background:** Breast cancer stem-like cells (BCSCs) are considered a source of tumor origins, metastasis and drug resistance, thereby limiting current treatment regimens. Stigmasterol has been reported to inhibit various cancer processes, but its effects and mechanisms in BCSCs have not been investigated.

**Methods:** To generate spheroids, we enriched parental and SUM159 cells with BCSCs in a serum-free medium. The effects on the stemness, metastasis and drug resistance of CSC-enriched SUM159 cells were detected for the first time by *in vivo* and *in vitro* experiments.

**Results:** CSC-enriched SUM159 and 4T1 cells demonstrated higher potential for tumorigenesis and metastasis. Stigmasterol suppresses BCSCs' spheroid formation, cell viability, and migration ability and promotes cell apoptosis. Stigmasterol also inhibited BCSCs-originated cancer formation in rat models. Stigmasterol also attenuated the growth of TNBC organoids from human breast cancer tissues. These data revealed the inhibitory effects of stigmasterol on BCSC traits. In the meantime, we found that JAK3 was upregulated in BCSCs, and Stigmasterol could effectively inhibit its expression. In addition, JAK3 was evidenced to negatively regulate BCSC activity and stemness both *in vitro* and *in vivo*. More importantly, the results indicated that Stigmasterol suppresses BCSC activity by inhibiting JAK3 expression.

**Conclusion:** This study is the first to demonstrate that Stigmasterol inhibited metastasis and stemness of BCSCs by downregulating JAK3, which might provide a new method for the clinical application of Stigmasterol in breast cancer.

## Introduction

Breast cancer is one of the most common malignant tumors and is the leading cause of cancer-related deaths among women worldwide 1, 2. The occurrence and development of breast cancer are usually caused by abnormal alterations in the pathway of cell proliferation regulating, differentiation, and apoptosis. It has been established that tumor growth and recurrence are regularly triggered by cancer stem cells (BCSCs); they are a small number of tumor-initiating cells; these cells possess robust self-renewal and metastasis ability and show strong resistance to standard chemotherapy 3, 4. These characteristics of BCSCs result in drug resistance and easy relapse. Therefore, inhibiting the activity of BCSCs has a significant influence on the treatment of breast cancer.

Although there is a small proportion of BCSCs in the entire tumor, plenty of methods have been developed to grow them in large quantities *in vitro* 5-7. Under appropriate growth conditions, cancer cells can grow in spheres. Compared with cells grown in monolayer culture, these spheroid-forming cells displayed altered cell surface markers and owned stem cell-like properties 8, 9. These spheres have been widely used to determine the characteristics of cancer stem cells both *in vitro* and *in vivo*, then evaluate the inhibitory activity of cytotoxic compounds on cancer stem cells 10, 11. The self-renewal behavior of BCSC involves multiple signaling pathways, including Wnt (Wingless-Int), Notch and Hedgehog pathways 12-14. Because BCSCs are resistant to traditional chemotherapy and radiotherapy, it is necessary to design new drugs and new therapies to target BCSCs in breast cancer. Interestingly, many compounds have been discovered to target BCSCs recently, such as curcumin 15, piperine 16, and sulforaphane 10. However, factors like weak dose-response and toxicity have primarily limited its applications. Therefore, it is significant to identify novel dietary compounds that can inhibit the activity of BCSCs and have low cytotoxicity.

Under proper culture conditions, BCSCs can grow in the form of spheres. Compared with cells grown in monolayer, these spheroid-forming cells exhibited altered cell surface markers; CD44 was commonly present in the cell membrane, while CD24 was regularly absent. Thus, CD44^+^/CD24^-^ cells in breast cancer cells were considered BCSCs, and CD44^+^/CD24^-^ cells were isolated by flow cytometry from breast cancer cells that exhibited stem cell-like properties 5. The self-renewal behavior of BCSC involves multiple signaling pathways, including Wnt (Wingless-Int), Notch and Hedgehog pathways 12-14, and the regulators involved in these pathways, such as Oct4, Nanong, C-Myc and Sox2, are commonly found upregulated in BCSCs and regularly used as markers of BCSCs 6, 7. Because BCSCs are resistant to traditional chemotherapy and radiotherapy, it is highly urgent to develop new drugs and therapy methods to target BCSCs in breast cancer. Interestingly, many compounds have been discovered to regulate BCSC activity recently, such as curcumin 15, piperine 16, and sulforaphane 10. Nevertheless, multiple factors, such as toxicity and weak dose-response, have already been strictly limited in the applications. Therefore, it is significant to identify novel dietary compounds that can inhibit the activity of BCSCs and have low cytotoxicity.

JAK3 is a non-receptor tyrosine kinase related to the signal transduction of the common gamma chain subfamily of cytokine receptors, mainly expressed in hematopoietic cells 17. Therefore, JAK3 is critical to the hematopoietic function and T cell development 18. JAK3 inactivating mutations cause immunodeficiency syndrome (SCID) 19. Recent studies indicated that abnormal activation of JAK3 was also associated with human malignancies. For example, JAK3 was constitutive activated in colon carcinoma tumors and inhibiting JAK3 expression leads to apoptosis and cell cycle arrest in colon carcinoma cells 20; JAK3 was aberrantly upregulated in non-small cell lung cancer cells and associated with cancer progression 21. However, its role in breast cancer is elusive.

Plant sterols have various pharmacological activities, such as antidiabetic, anti-inflammatory, and anticancer 22, 23. In addition, previous reports indicated that phytosterol-rich foods help to decrease the risk of developing cancer by 20% 24. Stigmasterol ((C29H48O), SS) is a common phytosterol rich in various vegetables. In recent years, SS has attracted attention due to its low cytotoxicity and anti-cancer properties. For example, Stigmasterol could inhibit Akt/mTOR pathway and simultaneously protect autophagy and induce apoptosis in gastric cancer cells 25; Stigmasterol inhibits endometrial cancer progression by repressing Nrf2 signal pathway 26; Stigmasterol inhibits cholangiocarcinoma growth and suppresses tumor angiogenesis by downregulating tumor necrosis factor-α 27. Until now, it is still unknown whether stigmasterol plays a role in breast cancer progression. This article studied the anti-CSC effect of stigmasterol and its potential molecular mechanism *in vitro* and *in vivo*.

## Methods

### Cell culture and transfection

SUM159 and 4T1 cells purchased from Cell Bank of Type Culture Collection Center of Chinese Academy of Sciences (Shanghai, China), cells were cultured in DMEM (Gibco, Grand Island, NY, USA) added with 2 mM L-glutamine, 10% FBS, 100 ml streptomycin and 100 ml penicillin. The isolated BCSCs were cultured in a serum-free RPMI1640 medium that contained bFGF, EGF, and B27 factors (Gibco, Grand Island, NY, USA). JAK3-pcDNA 3.1, pcDNA 3.1 vector, JAK3-shRNA-psi-U6^TM^, and psi-U6^TM^ vector were purchased from Beyotime Co., Ltd. (Shanghai, China), they were transfected into cells using Lipofectamine 3000 kit (Life Technologies, USA) according to the manufacturer's instructions.

### BCSC isolation

BCSC isolation was conducted as previously reported 8. Briefly, SUM159 and 4T1 cells were sorted into two sub-populations based on cell surface markers CD44 and CD24: cells with CD24-/CD44+ (BCSCs) and non-CD24-/CD44+ (non-BCSCs). SUM159 and 4T1 cells were trypsinized, suspended in the single-cell mixtures and washed with PBS after incubating with a monoclonal antibody specific for human cell surface markers CD44 (Ab23396, Abcam) and CD24 (Ab25494, Abcam) (Abcam, CAMB, UK) on ice for 30 minutes. These cells were collected, and then the FACS Calibur flow cytometer (BD Biosciences, Franklin Lakes, New Jersey, USA) and Cell Quest Pro software (BD Biosciences) were used for analysis.

### Immunocytochemistry

To check cell surface markers CD24 and CD44 expressions in SUM159 and 4T1 isolated BCSCs. These cells were fixed in 4% paraformaldehyde (-20°C, 10 minutes) and then permeabilised in PBST (PBS containing 0.05% Tween -20) and 0.1% triton X-100 for 10 minutes. Then, these cells were incubated at room temperature for 3 hours with PE-conjugated CD24 or PE-conjugated CD44 antibody (1:50) and stained with DAPI (1μg/ml). Finally, these cells were mounted for imaging on slides with Fluoromount G.

### RT-qPCR

Trizol reagent (Invitrogen, USA) was used to extract total RNA from corresponding cells. The extracted RNA was reverse transcribed into cDNA using an M-MLV first-strand cDNA synthesis kit (Omega, USA). Subsequently, the cDNA was used to perform a quantitative PCR assay with the following primers: GAPDH, GCAGTGFCA AAGTGGAGATTG (forward), and GCAGAAGGGGCGGAGATGAT (reverse); JAK3: TGTAAAACGACGGCCAGT (forward), AGGAAACAGCTATGACCATG (reverse). (2-ΔΔCT) method was used to determine the relative quantity of these mRNAs as described previously 25.

### Western blot

RIPA lysis buffer (Kangwei Century Biotechnology, Beijing, China) was used to extract the total protein of the cells, supplemented with protease and phosphatase inhibitors. These Proteins' concentration was determined with a BCA protein kit (TransGen Biotech, Beijing, China), and 10 ug of protein per well was loaded. These proteins were separated by 10% sodium dodecyl sulfate-polyacrylamide gel electrophoresis (SDS-PAGE) and transferred to polyvinylidene fluoride (PVDF) membranes. After being blocked by 5% nonfat dry milk and washed with TBST, these membranes were incubated with primary antibodies (1:1000) at 4 °C overnight and then secondary antibodies (1:5000) at room temperature for 2h, respectively. Finally, a Multifunctional Imaging Analysis System (Bio-Rad, Hercules, CA, USA) was applied to observe the chemiluminescence. The antibodies were purchased from Abcam Company), including JAK3 (ab45141, Abcam), Y785 phosphorylated JAK3 (Y785, p-JAK3) (ab61102, Abcam), Oct4 (ab181557, Abcam), Nanong (ab109250, Abcam), C-Myc (ab32072, Abcam), Sox2 (ab92494), and GAPDH (ab8245, Abcam).

### MTT assay for cell viability

For the MTT assay, a density of 1x10^4^ cells/ml BCSCs cells was seeded onto 96-well plates containing 100 µl RPMI-1640. After 8 h, these cells were treated for 48 h with various concentrations of Stigmasterol (0, 1, 5 and 10 μM). Then, 20 µl MTT (5 mg/ml, 20 µl/well) was added to each well and then incubated for 4 h. Afterwards, the supernatant from each well and the bottom cells were resolved with DMSO (150 µl per well). Cell density was measured using an ELX800-UV absorption microplate reader (BioTek Instruments Co., Ltd., Winooski, VT, USA).

### Transwell for migration and invasion assays

The transwell assay was performed as in the previous report 16. Approximately 2×10^4^ cells were inoculated in the upper chamber of the transwell assay platform for 24 hours to allow them to migrate to the lower chamber. Crystal violet (0.2%) fixed and stained the transwell membranes. The cells on the lower surface of the Millipore membrane (EMD Millipore Corporation, Billerica, MA) were counted by an optical microscope at 200× magnification.

### Spheroid formation assay

BCSCs were treated with 0, 1, 5 and 10 μM Stigmasterol in six-well plates for 24 h. Then single-cell suspensions were prepared on six-well ultralow attachment plates (Gibco, Grand Island, NY, USA) at 1000 cells/ml density. These cells were grown in DMEM containing 1% N2 (Sigma, St. Louis, Missouri, USA), 2% B27 (Sigma), 100 ng/ml epidermal growth factor (Invitrogen), 1% antibiotic-antimycotic (Invitrogen, Waltham, MA, USA) and 20 ng/ml human platelet growth factor (Sigma). After 7 days of culturing, the mammospheres above 50.0 mm diameter were counted.

### *In vivo* mouse experiments

JAK3 were sub-cloned into a pCMVRed-Firefly-Luc plasmid, and the lentivirus was packed in 293T cells. PCMV-Red-Firefly-Luc vector was set as a negative control. BCSCs were infected by the harvested lentivirus (MOI D10). After the selection with Doxycycline (2mg/ml) for two weeks, 2 × 10^6^ BCSCs which stably expressed JAK3 were injected into female BALB/C nude mice, and the empty PCMV-Red-Firefly-Luc vector was set as control. Then, these mice were mammary fat pad injected with a dosage of 10 mg/kg Stigmasterol (200 µg/100 µL) or DMSO (as control) once a week for 1.5 months. After this, the tumor volume was measured using Bioluminescent IVIS imaging system 200 (Xenogen CA, USA). At day 80, these mice were sacrificed, and tumor tissues were used to detect JAK3 expression. Animal care and animal experiments were conducted using protocols approved by the Animal Care and Use Committee of Ethics Guangdong Provincial Hospital of Chinese Medicine (approval number ZE2020-334-01). Female BALB/C nude mice (5 weeks old) were obtained from Shanghai Model Organisms Center, Inc. (Shanghai, China) and kept in mouse facilities for 7 days before the indicated experiments.

### Organoid culture

Breast cancer and corresponding non-tumor normal tissues were obtained from TNBC patients in Guangdong Provincial Hospital of Chinese Medicine, and Ethics Committees have approved this study. Based on previous studies 28, 29, Organoids were cultured using the human breast cancer organoid kit (Biorgen, Guangzhou, China). Briefly, tissues were cut into small pieces and incubated with tissue digestion solution in basal medium for 1 h at 37°C. After resuspension, the cell suspension was centrifuged and treated with erythrocyte lysis buffer for 5 min. After washing, the cells were incubated on pre-warmed tissue culture plates with 8-10 mg /ml matrigel (Mogengel, Xiamen, China) at 37°C for 15 min and supplemented with a complete medium. Organoids were passaged (15-30 days) with TrypLE™ (Thermo Fischer).

### H&E staining

The organoids were collected, fixed with 4% paraformaldehyde, dehydrated with gradient ethanol, and transparently treated with xylene for 5 min. After paraffin embedding, the organoids were sliced (approximately 5 μm thick). The organoid slices were baked on a film dryer to increase the adhesion of the organoids. The organoid sections were sequentially immersed in xylene 3 times, 100% ethanol 3 times, 95% ethanol 3 times, each time for 3 min, and ultrapure water for 3 min. 40 μL of hematoxylin dye was added dropwise to the organoids for 10 s. After rinsing, the sections were treated with hydrochloric acid alcohol and sodium bicarbonate for bluing and immersed in eosin for 10 s. The sections were sealed with neutral resin after the gradient ethanol and xylene treatments. The sections were observed and photographed under a light microscope.

### Immunohistochemistry (IHC)

After baking and dewaxing, the sections were subjected to antigen repair and transferred to 0.3% Triton X-100 for 20 min. After PBS washing, the sections were blocked for 45 min by dropwise addition of 40 μL of goat serum and incubated with 40 μL of diluted primary antibodies, including estrogen receptor (ER; Abcam; 1: 50), human epithelial growth factor receptor-2 (Her2; Abcam; 1: 50), progestogen receptor (PR; Abcam; 1: 50) overnight at 4°C. After washing, the sections were incubated with 40 μL of horseradish peroxidase-conjugated secondary antibody (Abcam; 1: 50) for 1 h. After washing, the sections were incubated with a drop of DAB solution. When the organoids were brown, they were quickly rinsed under running water for about 3 min. Hematoxylin dye was added for nuclei staining for about 10 s. After washing, the sections were put into hydrochloric alcohol and sodium bicarbonate for 1 min. After washing, the sections were treated with gradient alcohol and xylene and sealed with neutral resin. Photographs were taken for observation under a light microscope, and the staining results were analyzed.

### Statistical analysis

Data were analyzed by SPSS 10.0 software. At least three independent experiments were conducted for each assay. All data are demonstrated as the mean ± SD. A one-way analysis of variance (ANOVA) followed by a student t-test was used in the present study. *p* < 0.05 was considered statistically significant.

## Results

### Stigmasterol suppresses the CD44^+^CD24^-/low^ cancer stem cell-like immunophenotype in breast cancer cells

**Figure 1:**
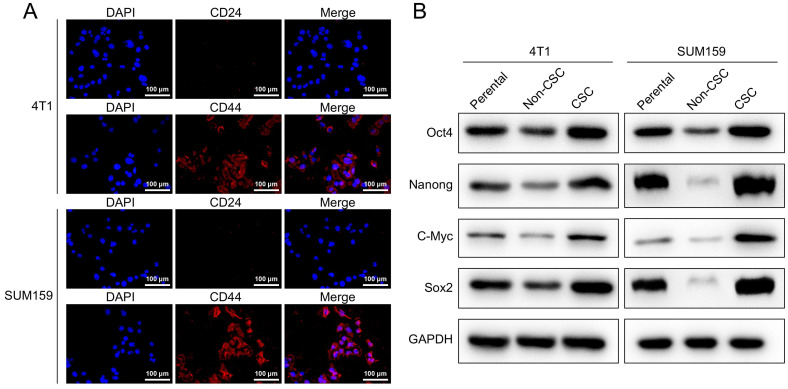
**Characterization of breast cancer stem-like cells. (A)** An immunocytochemistry assay was performed to detect the expression of cell surface markers CD44 and CD24 in SUM159, and 4T1 isolated breast cancer stem-like cells (magnification 200 ×). **(B)** Western-blot was carried out to examine the expression of BCSCs markers, including Oct4, Nanong, C-Myc and Sox2 in SUM159 or 4T1 cells (Parental), non CD44+/CD24- cells (Non-CSC), and CD44^+^/CD24^-^ cells (CSC). GAPDH was used as an internal control.

CD44 and CD24 are cell surface glycoproteins that play critical roles in cell adhesion and migration. Previous studies have shown that breast cancer cells contain stem-cell-like cells that express CD44+CD24-/low marker, and compared to non-CD44+CD24-/low cancer cells, these cells possess more than 50-fold increased tumorigenic capacity. To study the effect of Stigmasterol on breast cancer stem cells, we established model stem cell-like spheroids from two types of breast cancer cell lines 4T1 and SUM159 with stem properties marker CD44^+^CD24^-/low^ by flow cytometry and determined the expressed CD44 and CD24 in these selected breast stem cells with Immunofluorescence assay, as shown in Fig. 1A, CD24 remains low level and CD44 exhibits high level in these cells. In addition, we examined the expression of stem properties markers, including Oct4, Nanong, C-Myc, and Sox2, to determine the stemness of these cells. These proteins' levels were markedly upregulated in the sorted cells compared to their parental and non-BCSCs. These results indicated that BCSCs were successfully selected from MCF-7 and MDA-MB-231, and these cells were used for the following experiments in this study.

**Figure 2:**
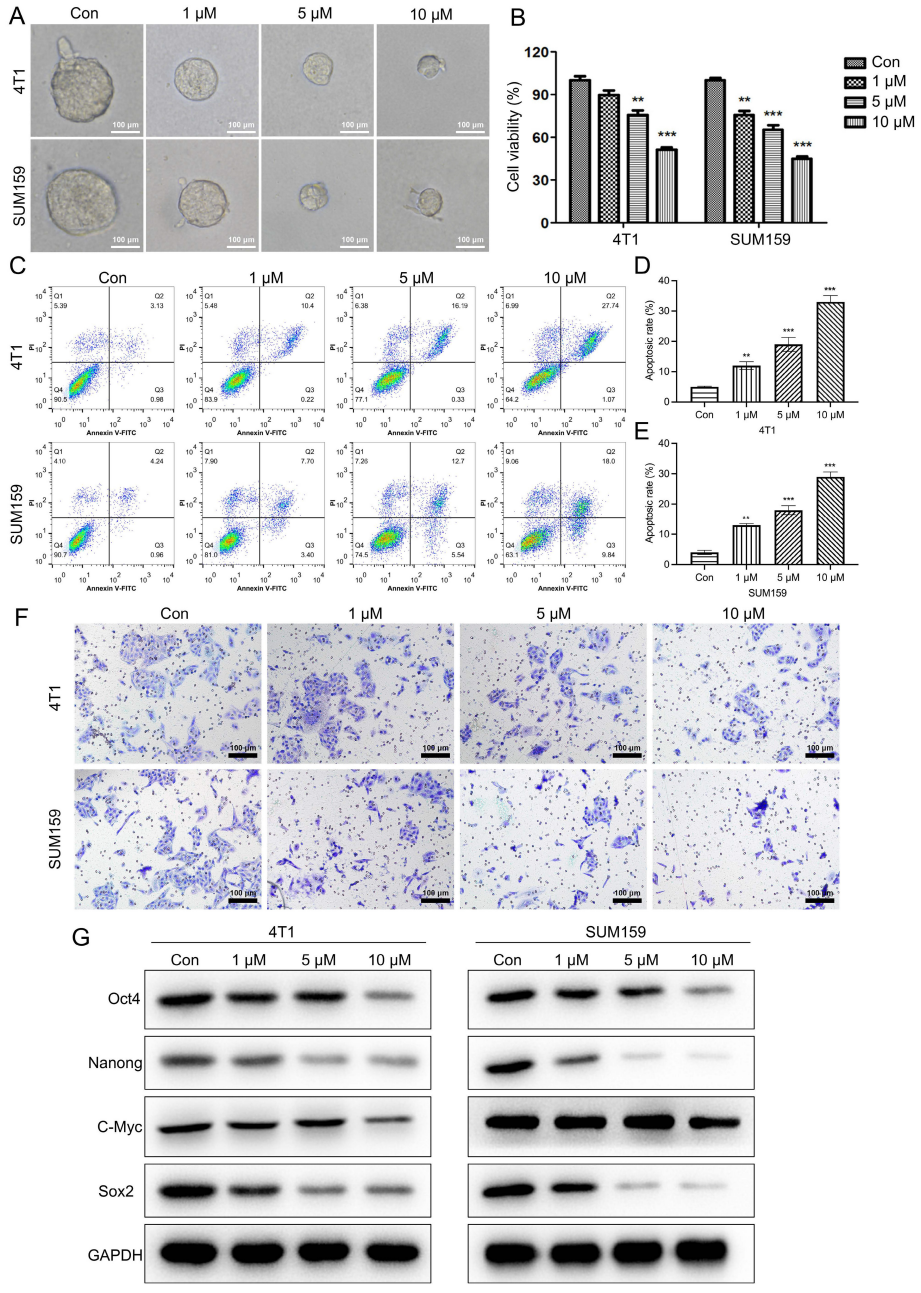
** Stigmasterol inhibits the stemness of BCSCs.** SUM159 or 4T1 isolated BCSCs were pretreated with 0, 1, 5, or 10 μM Stigmasterol for 24 h, then **(A)** Spheroid formation assay (magnification 200 ×), **(B)** MTT assay, **(C)** Flow cytometry, and **(F)** Transwell assay (magnification 200 ×) were performed to detect the spheroid forming ability, cell apoptosis, cell viability and migration ability, respectively. (D, E) statistical result of **(C)**. **(G)** A western blot was performed to detect the expression of BCSC markers, including Oct4, Nanong, C-Myc and Sox2, in BCSCs isolated from SUM159 or 4T1 cells. **p<0.01, ***p<0.01.

As indicated in Fig. 2A, the size of the mammospheres was dramatically decreased following Stigmasterol administration (Fig. 2A). Further, the cell viability of BCSCs was determined, as verified by MTT assay, Stigmasterol dose-dependently inhibits the cell viability (Fig. 2B). Furthermore, the effect of Stigmasterol on the apoptosis and migration properties of BCSCs were examined using Flow cytometry and Transwell assay, respectively. As evidenced by Fig. 2C-F., the cell apoptosis of both 4T1 and SUM159 sorted BCSCs was significantly promoted by Stigmasterol, and the migration ability of these cells was dose-dependently inhibited following Stigmasterol treatment. Meanwhile, Stigmasterol treatment also downregulated Bcl-2 in BCSCs isolated from SUM159 and 4T1 cells (Supplementary Fig. 1). These results verified that Stigmasterol could effectively inhibit the activity of BCSCs. Moreover, we explore the effect of Stigmasterol on the stemness of BCSCs by detecting the expression of stem cell markers (Oct4, Nanong, C-Myc, and Sox2) using western-blot, as indicated in Fig. 2G, the expression level of these proteins was all dose-dependently inhibited by Stigmasterol, suggested that Stigmasterol suppress the stemness of BCSCs.

### JAK3 negatively regulates the activity of BCSCs

**Figure 3:**
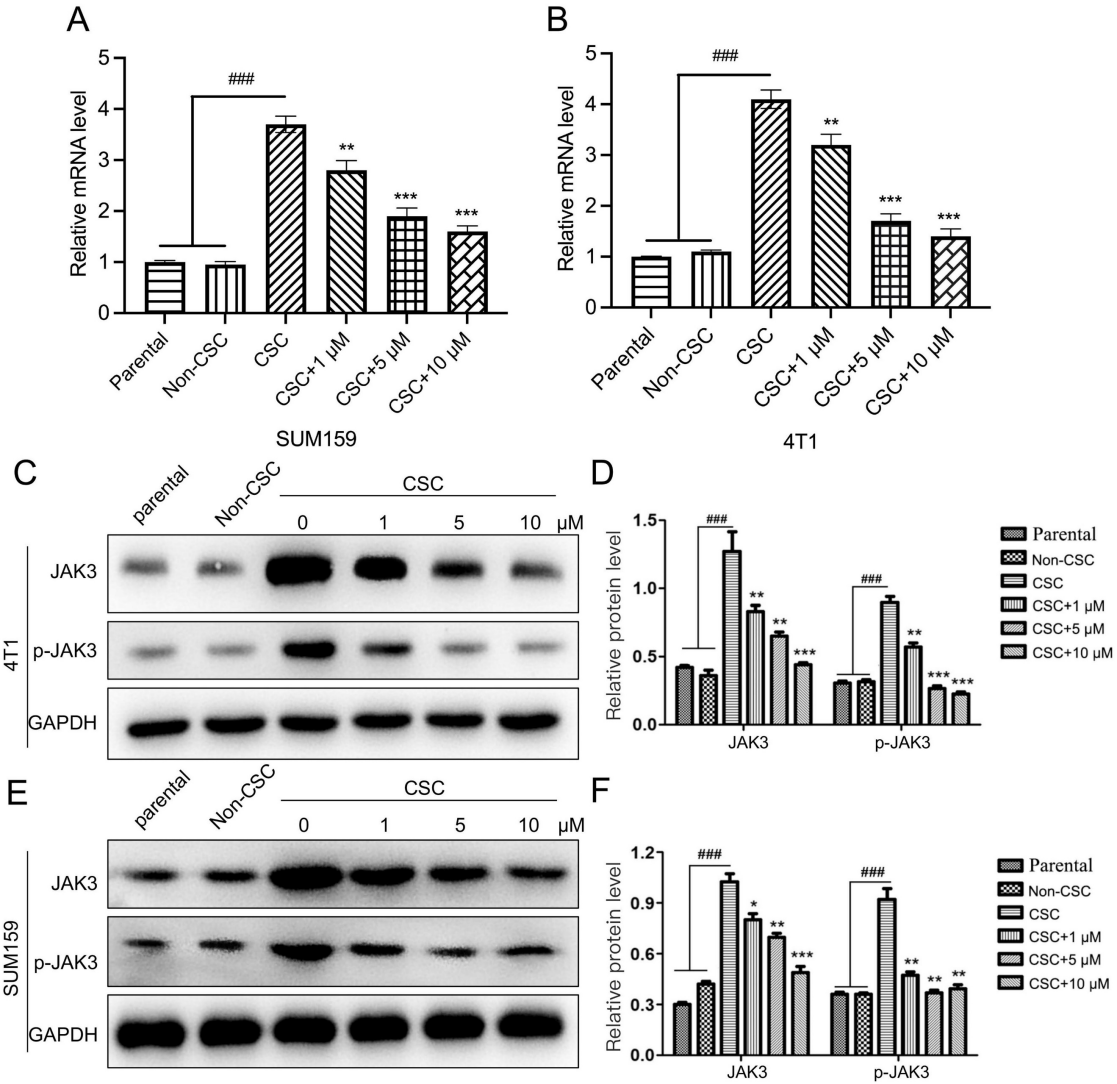
**Stigmasterol inhibits JAK3 expression in BCSCs. (A, B)** qRT-PCR was carried out to examine the mRNA level of JAK3 in 4T1 **(A)** or SUM159 **(B)** parental, Non-CSC, or CSC cells that were pretreated with 0, 1, 5, or 10 μM Stigmasterol for 24 h. GAPDH was used as an internal control. **(C, E)** Western blot was performed to detect JAK3, or phosphorylated JAK3 (p- JAK3) in these cells; GAPDH was used as the internal control. **(D, F)** statistical result of **(C)** and **(E)**. ^###^p<0.001; *p<0.05, **p<0.01, ***p<0.01.

JAK3 was regularly found upregulated in various cancer cells, such as colon and non-small cell lung cancer (19, 20). However, its function in breast cancer is elusive. Therefore, we explored its role in BCSCs. We first determined JAK3 expression in 4T1 and SUM159 and the corresponding BCSCs using RT-PCR and western-blot assays. As indicated in Fig. 3A, B, the JAK3 mRNA level in BCSCs was upregulated. Meanwhile, the phosphorylated levels of JAK3 (the activated JAK3) and total JAK3 were dramatically elevated (Fig. 3C-F). These results demonstrated that JAK3 may play a role in regulating BCSC activity.

**Figure 4:**
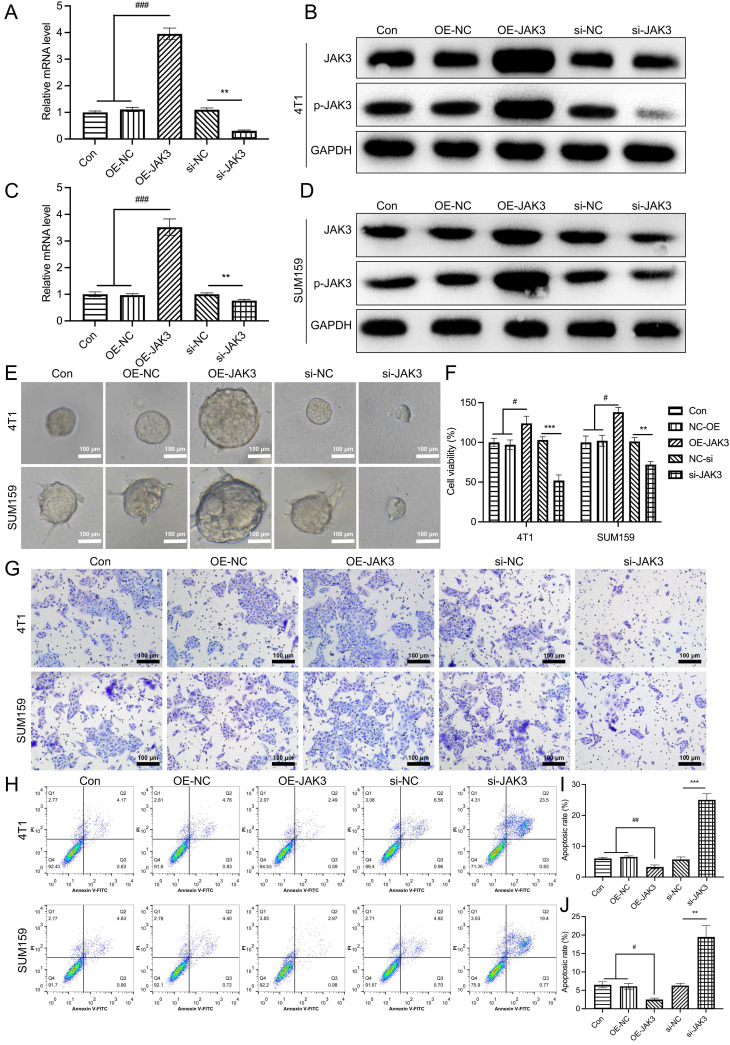
** JAK3 promotes the stemness of BCSCs**. SUM159 or 4T1 isolated BCSCs were transfected with empty pcDNA3.1 vector (OE-NC), JAK3-pcDNA3.1 (OE-JAK3), empty vector psi-U6^TM^ (si-NC) or JAK3-shRNA-psi-U6^TM^ (si-JAK3), and 48 h after the transfection, mRNA **(A, C)** and protein **(B, D)** levels of JAK3 and p-JAK3 were determined with qRT-PCR and Western-blot assay, respectively. GAPDH was used as an internal control. **(E)** Spheroid formation assay (magnification 200 ×), **(F)** MTT assay, **(G)** Transwell assay (magnification 200 ×), and **(H)** Flow cytometry assays were performed to detect the spheroid forming ability, cell viability, migration ability, and cell apoptosis, respectively. **(I, J)** statistical result of **(H)**. *p<0.05, **p<0.01, ***p<0.01.

To further determine its role in BCSCs, JAK3 was overexpressed and knocked down in these BCSCs, as shown in Fig. 4A-D. The mRNA and protein levels (total and phosphorylated levels) of JAK3 were successfully manipulated in these cells. Subsequently, we examined their effect on cell mammosphere, viability, and migration. As indicated in Fig. 4E-J. The sphere formation ability of JAK3 overexpressed BCSCs was significantly enhanced in contrast with that in control, while this ability was markedly inhibited in JAK3 knockdown BCSCs (Fig. 4E). Meanwhile, we found the cell viability (Fig. 4F) and migration capability (Fig. 4G) was dramatically promoted in BCSCs that with JAK3 overexpression but decreased in these cells that with JAK3 knocked down as compared with the control. Furthermore, we determined JAK3 function on the apoptosis of these BCSCs (Fig. 4H-J); the cell apoptosis of both 4T1 and SUM159 sorted BCSCs was significantly inhibited by JAK3 knockdown but promoted by JAK3 overexpression. These results indicated that JAK3 was upregulated in BCSCs and regulates the activity and stemness of BCSCs.

### Stigmasterol suppresses BCSC activity by inhibiting JAK3 expression

**Figure 5:**
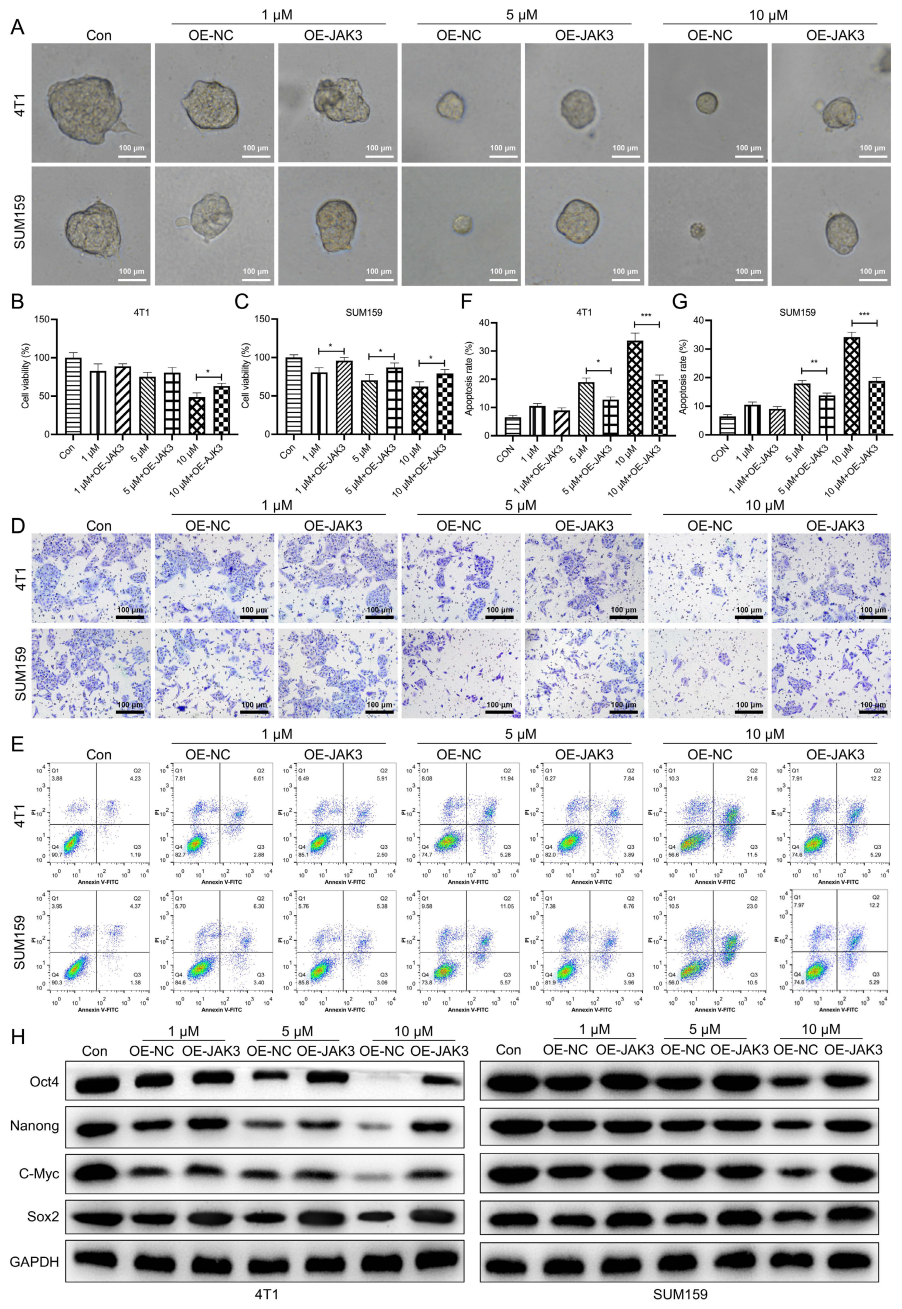
** JAK3 overexpression suppresses the inhibition effect of Stigmasterol on BCSCs**. After SUM159 or 4T1 isolated BCSCs were transfected with OE-NC or OE-JAK3 for 48 h, these cells were treated with 0, 1, 5, or 10 μM Stigmasterol for 24 h. Then **(A)** Spheroid formation assay (magnification 200 ×), **(B, C)** MTT assay, **(D)** Transwell assay (magnification 200 ×), and **(E)** Flow cytometry assays were performed to detect the spheroid forming ability, cell viability, migration ability, and cell apoptosis, respectively. **(F, J)** statistical result of **(E)**. **(G)** Western blot was performed to detect the expression of BCSC markers, including Oct4, Nanong, C-Myc and Sox2 in these cells; GAPDH was used as the internal control. *p<0.05, **p<0.01, ***p<0.01.

As JAK3 was upregulated in BCSCs and the RNA-Seq result indicated that Stigmasterol suppresses JAK3 expression in BCSCs, Stigmasterol may perform its inhibitory function in BCSCs by suppressing JAK3 expression. The functional interaction between Stigmasterol and JAK3 on BCSC activity was determined to test this speculation. As shown in Fig. 5A-C. 5 and 10 μM Stigmasterol inhibited BCSCs cell atmosphere, both effectively reversed by JAK3 overexpression. In the meantime, we found that Stigmasterol dose-dependently inhibited BCSCs viability and migration and promoted cell apoptosis, partially reversed by JAK3 overexpression (Fig. 5D-G). Moreover, we determined the stemness in these cells, and as verified in Fig. 4D, the inhibitory effect of Stigmasterol on Oct4, Nanong, C-Myc, and Sox2 expression was by JAK3 overexpression. These results together indicated that Stigmasterol inhibits BCSC activity and stemness by suppressing JAK3 expression.

### Stigmasterol suppresses BCSC activity by inhibiting JAK3 expression *in vivo*

**Figure 6:**
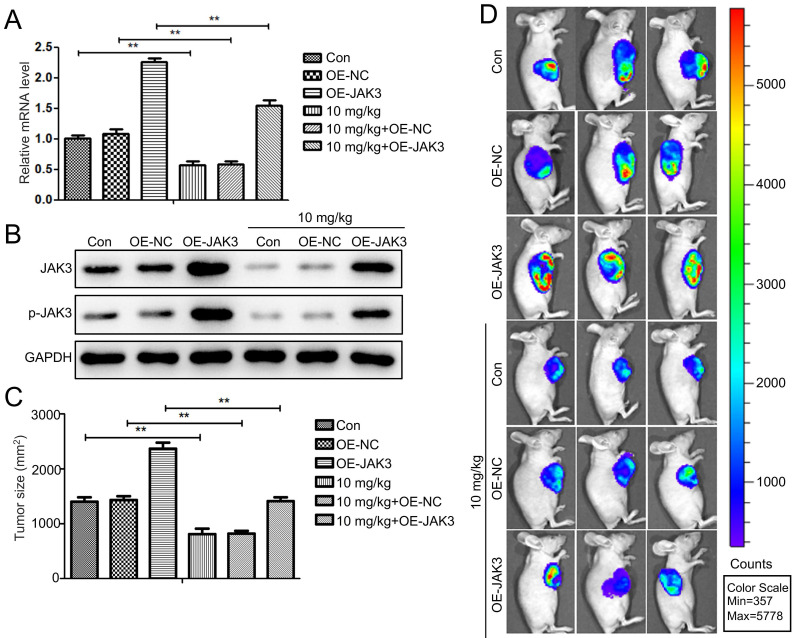
** JAK3 overexpression attenuates the inhibition effect of Stigmasterol on BCSCs* in vivo*. (A)** qRT-PCR was conducted to examine the mRNA level of JAK3 in OE-NC or OE-JAK3 transfected tumor tissues in mice administered with or without 10 mg/kg Stigmasterol. **(B)** Western blot was performed to detect the expression of JAK3 and p-JAK3 in these tumor tissues, and GAPDH was used as an internal control. **(C)** The tumor volume of OE-NC or OE-JAK3 transfected tumor tissues in mice administered with or without 10 mg/kg Stigmasterol was measured using a Bioluminescent IVIS imaging system 1.5 months after the cell injection. **(D)** Statistical result of **(C)**. **p <0.01.

To further investigate the impact of Stigmasterol and JAK3 expression on the tumor formation ability of BCSCs *in vivo*, we first established the JAK3 stably overexpressed BCSCs (4T1 sorted BCSCs). The successful manipulating of JAK3 expression in BCSCs was shown in Fig. 6A, B. IVIS images of mice at day 42 were demonstrated in Fig. 6C, D. NOD mice in NC group inoculated with 2×10^6^/ml cells into fat pad formed 1.5 cm diameter tumors in day 42. In contrast, those in the JAK3 overexpressed group formed 2.3 cm diameter tumors, suggesting JAK3 promotes the tumor formation ability of BCSCs *in vivo*. Further, we determined the effect of Stigmasterol on BCSCs tumor formation ability, compared with the control group, 10mg/kg Stigmasterol administration significantly inhibited the tumor formation (0.8 cm diameter tumors in Stigmasterol treated group VS 1.5 cm diameter tumors in the control group) (Fig. 6C, D), indicated that Stigmasterol inhibits tumor formation ability of BCSCs *in vivo*. More importantly, it was evidenced that Stigmasterol inhibited JAK3 expression in tumor tissues, and the inhibitory effect of Stigmasterol on tumor formation was partially reversed by JAK3 expression. These results together indicated that Stigmasterol suppresses BCSC activity by inhibiting JAK3 expression *in vivo*.

### Stigmasterol inhibits the growth of TNBC organoids

**Figure 7:**
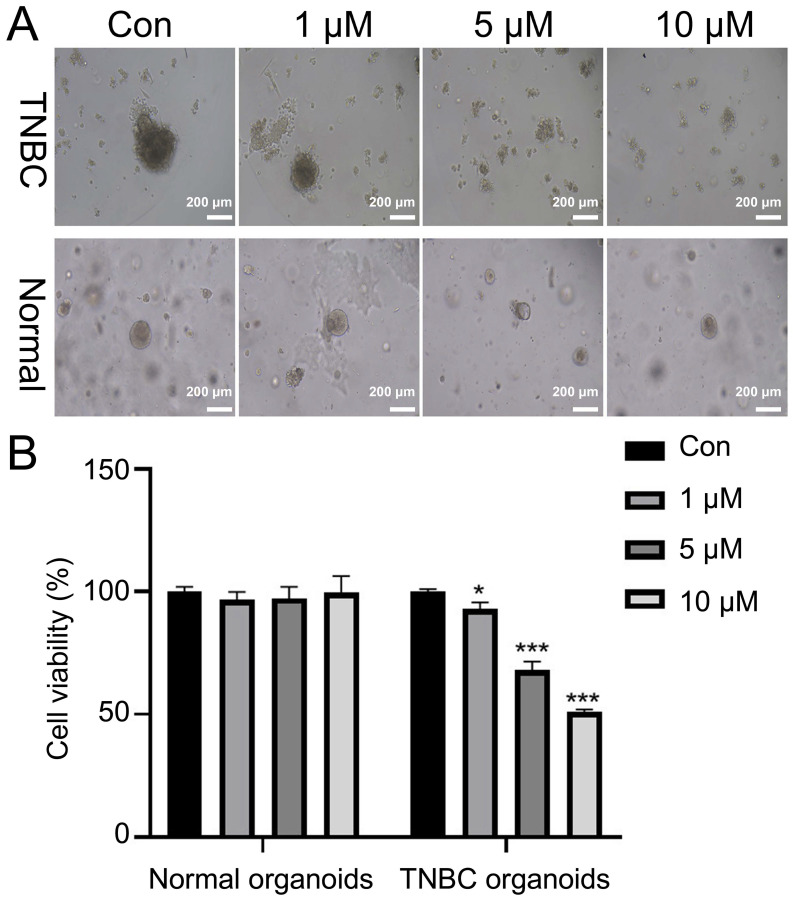
** Stigmasterol inhibits cell viability of TNBC organoids. (A)** After treatment with 0, 1, 5, and 10 μM stigmasterol, the morphology of the organoids was observed (magnification 100 ×). **(B)** CCK-8 determined the cell viability of the organoids. *p <0.05, ***p <0.001.

Next, the TNBC organoid was cultured from breast cancer tissues of TNBC patients. H&E staining results indicated that the nuclei of TNBC organoids were blue-purple, and the arrangement of cells was disordered with glandular cavity structure, consistent with the morphology and distribution of breast cancer cells. At the molecular level, we used IHC to analyze the levels of three representative molecular markers (ER, PR and HER2) in breast cancer. The results showed that Ki67 was strongly expressed and ER, PR and HER2 were weakly expressed in breast cancer cells. The intensity of Ki67, ER, PR and HER2 expression in constructed organoids was similar to that of their breast cancer (Supplementary Fig. 2). Microscopic results showed that stigmasterol treatment markedly reduced the size of TNBC organoids, especially 5 and 10 μM stigmasterol (Fig. 7A). Besides, CCK-8 data denoted that stigmasterol treatment could decrease the cell viability of TNBC organoids. The reduction effect was dose-dependent (Fig. 7B).

## Discussion

Breast cancer has become one of the most common cancers in the world due to its character of substantial morbidity and mortality 30. The chemotherapeutic drugs for breast cancer currently have adverse effects in clinical practice. Therefore, exploring new effective anticancer drugs with fewer side effects is urgent. Stigmasterol is the principal plant sterol in dietary plants, which has minimal side effects and is low-cost 31. It is reported that Stigmasterol exhibited anticancer potential against some cancer cell lines 32-34, thus providing a new therapy for cancer treatment. However, few studies have been conducted on the effect of Stigmasterol on the activity of BCSCs and the potential molecular mechanisms in BCSCs. Here, our result for the first time indicated that Stigmasterol could inhibit BCSC activity and suppress the stemness of BCSCs by inhibiting JAK3 expression.

Many chemotherapeutic and anticancer drugs can modulate the activity of cancer stem cells in a series of cancers through different pathways 35. However, evidence on the role of Stigmasterol in breast cancer stem cells was still insufficient. The present study discovered Stigmasterol-induced inhibition in BCSC activity for the first time. The treatment of Stigmasterol could inhibit BCSC viability, migration, and sphere formation ability while promoting BCSC apoptosis. In addition, we found the stem cell marker genes OCT4 and Stigmasterol effectively inhibited SOX6.

Breast cancer organoid models are *in vitro* models derived from patients' tumor tissues with high *in vivo* heterogeneity 29. Studies have shown that the response of breast cancer organoid models to drugs matches well with that of the human body, and the consistency between the negative response of the organoid and the patient's body for the same drug is up to 100%, and the consistency of the positive response is up to 98% 36-38. Therefore, the establishment of breast cancer organoid models provides new ideas for drug screening, precision tumor therapy and individualized treatment. In this study, we constructed TNBC patient-derived organoids. We found that TNBC organoids have large nuclei and a high nucleoplasm ratio of tumor cell characteristics and maintain the same expression profile as ER, PR and HER. Our constructed TNBC organoid is consistent with the tumor tissue in terms of characterizing tissue morphology, cellular composition, and marker expression and can be used for subsequent experiments. Additionally, our data denoted that stigmasterol could inhibit TNBC organoids' growth, further revealing stigmasterol's inhibitory effect in breast cancer progression from the clinical aspect.

JAK3 pathway, a vital signaling cascade, can be activated in various cancers, whose activation could promote cancer progression 39-41. JAK3 is a significant negative regulator in cancer cell viability and apoptosis by regulating various downstream factors, such as STAT3, Akt and PS6K. The continuous stimulation of JAK3 signaling plays a vital role in maintaining malignant tumors. Some anticancer drugs could induce autophagy and apoptosis by blocking the JAK3 signaling pathway in various cancer cells 42. Therefore, we speculate that the JAK3 pathway may also related to breast cancer and play a key role in Stigmasterol-inhibited BCSC activity. In this research, we discovered that the JAK3 pathway was involved in BCSC activity inhibition triggered by Stigmasterol, which was proved by the downregulation of JAK3 and p-JAK3. Furthermore, overexpression of the JAK3 signaling pathway could directly influence activation, and knockdown of JAK3 expression suppressed BCSC activity. In our study, JAK3 overexpression attenuated the inhibitory effect of SS on BCSC activity and stemness. These results indicated that Stigmasterol inhibits BCSC activity and stemness by suppressing the JAK3 expression. However, further mechanisms need to be detected in future studies.

Therefore, the present study demonstrated for the first time that stigmasterol-mediated JAK3 pathway has unique advantages and significant potential in the treatment of BCSCs. In terms of clinical application, future studies need to further explore the pharmacology and toxicology of stigmasterol and its combination with existing treatments to provide new strategies for the treatment of breast cancer.

## Conclusion

This study demonstrated that Stigmasterol could inhibit BCSC activity and stemness, and JAK3 promotes the activity and stemness of BCSCs both in the intro and vivo. Furthermore, our study showed that SS inhibits BCSC activity and stemness by suppressing the JAK3 expression (see graphical abstract). It would enrich our fundamental understanding of the antitumor properties of Stigmasterol, otherwise providing evidence that stigmasterol may become a potential anticancer drug treatment for breast cancer in the future.

## Supplementary Material

Supplementary figures.
