# Supplementary material for: Stigmasterol Attenuates Triple-negative Breast Cancer Stem Cell Properties by Inhibiting JAK3
Source: J Cancer. 2025 Feb 3;16(5):1618–30. doi: 10.7150/jca.94822 (PMC11843247; doi:10.7150/jca.94822)
Supplement: Supplementary file 1 — Supplementary figures. [file jcav16p1618s1.pdf]

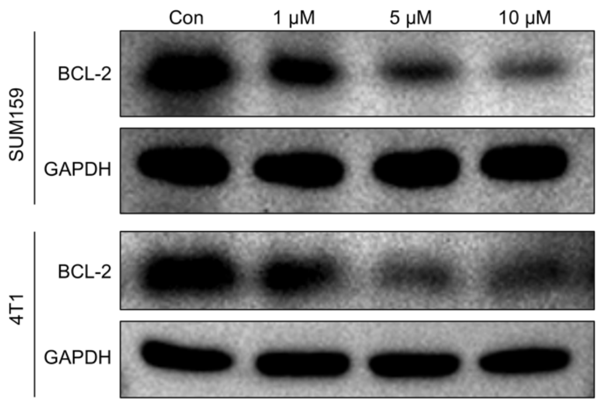

Supplementary Fig. 1. Stigmasterol downregulates Bcl-2 in BCSCs isolated from SUM159 and 4T1 cells. Western blotting analysis of Bcl2 in BCSCs after treatment with 0, 1, 5, and 10 μM stigmasterol.

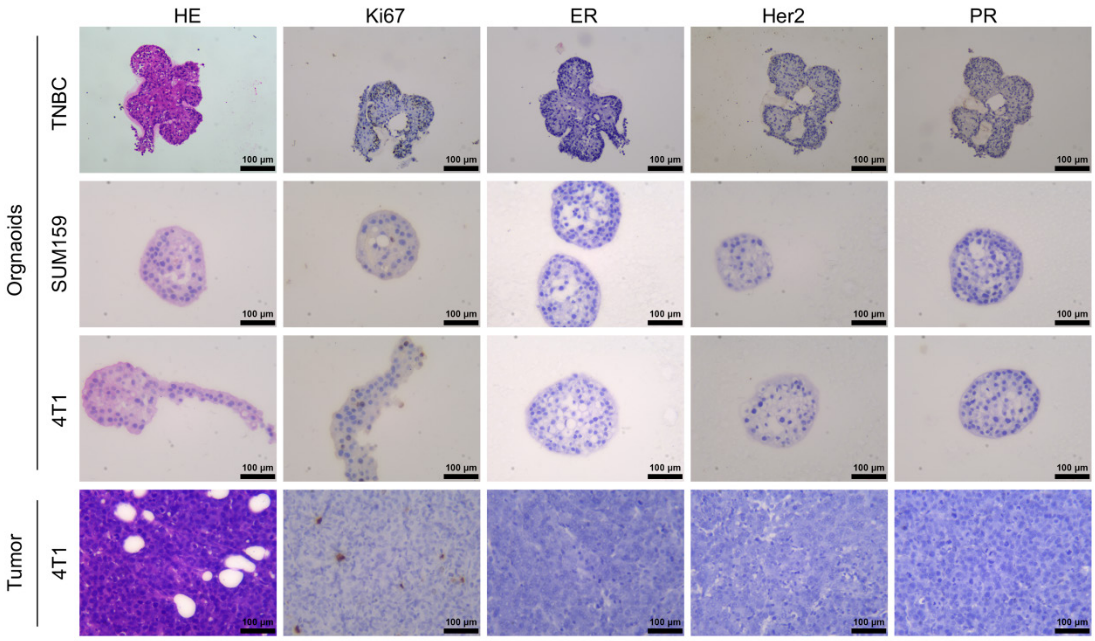

Supplementary Fig. 2. Identification of breast cancer cells and organoids. (A) Pathological structures were identified using H&E staining, and Ki67, ER, Her2, and PR expressions were assessed using IHC staining in breast cancer cells and organoids (magnification 200×).
